# SlideJ: An ImageJ plugin for automated processing of whole slide images

**DOI:** 10.1371/journal.pone.0180540

**Published:** 2017-07-06

**Authors:** Vincenzo Della Mea, Giulia L. Baroni, David Pilutti, Carla Di Loreto

**Affiliations:** 1 Department of Mathematics, Computer Science and Physics, University of Udine, Udine, Italy; 2 Department of Medicine, University of Udine, Udine, Italy; Medical University of Graz, AUSTRIA

## Abstract

The digital slide, or Whole Slide Image, is a digital image, acquired with specific scanners, that represents a complete tissue sample or cytological specimen at microscopic level. While Whole Slide image analysis is recognized among the most interesting opportunities, the typical size of such images—up to Gpixels- can be very demanding in terms of memory requirements. Thus, while algorithms and tools for processing and analysis of single microscopic field images are available, Whole Slide images size makes the direct use of such tools prohibitive or impossible. In this work a plugin for ImageJ, named SlideJ, is proposed with the objective to seamlessly extend the application of image analysis algorithms implemented in ImageJ for single microscopic field images to a whole digital slide analysis. The plugin has been complemented by examples of macro in the ImageJ scripting language to demonstrate its use in concrete situations.

## Introduction

The digitalization of full glass slides containing tissue samples for microscopic analysis is a reality that is becoming stronger and opens to many opportunities [1]. The digital slide, or Whole Slide Image, is a digital image, acquired with specific scanners, that represents a complete tissue sample or cytological specimen at microscopic level. Having such samples in digital form brings many advantages such as the immutability of the sample along time as well as the possibility to analyze it more automatically using many different image analysis methods. However, digital slides are acquired at resolutions up to 0.2 μ/pixel, thus consisting of up to billions of pixels. Image size is one of the reasons why they are not yet commonly used in routine.

While Whole Slide Imaging can be applied in any microscopy-based area, Pathology is the specialty with most attention to digital slides. As a matter of fact, under the term “digital pathology” now many digital slide applications are collected, including in particular telemedicine [2] and image analysis [3]. In particular, image analysis over digital pathology samples is aimed at tissue classification [4], quantification of biomarkers [5], identification of rare events like mitoses [6], etc.

ImageJ is a well known and long-lived open source software for biomedical image analysis [7]. It allows for the most important image processing and analysis procedures on a variety of image formats and sources, but it also allows for its extension and customization through add-ons such as macros and plugins. Macros (that is, interpreted software programs written in the internal scripting language) can be programmed for implementing specific functions not already available or to combine existing functions to be automatically executed. Furthermore, plugins (external software modules that can be run from a menu or inside macros) can be also implemented using the Java language, for faster and more complex functionalities. A large community of developers, which often are dedicated users with biological background, continuously enhances ImageJ by addressing bugs and particularly developing plugins and macros [8]. This eventually led to the birth of a project, “Fiji”, aimed at enhancing ImageJ architecture and update mechanism [9]. By means of its extensions, ImageJ may become a platform that can be specialized for usage in specific image processing and analysis areas. Among those areas, microscopy-originated images [10,11] have been one of ImageJ targets since the very beginning [12], although normally processing occurs on selected microscopic fields, instead of the whole slide.

The advent of digital slides is however posing one major issue, correlated with the size of the images which could be in the order of hundreds of Mpixels. ImageJ—and almost any other generic biomedical image processing software—is made for processing images that can be fully loaded in the main memory, with limitations coming from both the memory management side and the internal data structures (e.g., with a defined maximum number of pixels) since are not aimed at very large images. Processing large images can only be done by loading portions of them into memory, one at a time, until the complete image has been treated. In fact, digital slides image formats allow this by using tiles (i.e., small sub-images) as means to store the whole slide, at different magnifications according to a pyramidal scheme. Tiles can be individually extracted and eventually processed. Each format typically stores a number of image series (consisting in a group of tiles), each one representing a different magnification, or also a slide thumbnail, or even the label part of the slide. Tile size can be different among formats or even inside the same format.

Among ImageJ plugins, one notable contribution of interest for digital slides is Bio-Formats [13], a Java library for accessing many different biological image formats including a number of digital slide formats too. With Bio-Formats, one can read a full digital slide at a magnification sufficiently small to have a manageable image, or also extract crops at any magnification and of any (manageable) size. This allows to use ImageJ also for processing digital slides. Since in a number of algorithms the basic workflow is always the same (that is: extraction of a tile, execution of a procedure on it, iterated on the whole slide), one further abstraction step is to free the developer from the implementation of the common parts of this workflow.

Thus, the present paper describes the design, implementation and test of a plugin that, exploiting Bio-Formats basic input capabilities, enables to automatically run an ImageJ macro, possibly developed and tested on a single image, on a full digital slide.

In our knowledge only three works with similar aims but different implementation strategies are described in literature. Deroulers et al. [14] described an open source tool for splitting Hamamatsu digital slides (NDPI format) in tiles, that then could be processed with any other tool, including ImageJ. Nelissen et al. [15], with their SlideToolkit, implemented a similar workflow abstraction layer but based on the CellProfiler software [16]. A similar concept is proposed by Haak et al. [17], although again specialized on NDPI format, in the form of a ImageJ plugin that unfortunately is not available. Our proposal allows flexibility regarding the input image format, while directly focusing on ImageJ, with the possibility of exploiting everything from it through macros.

## Material and methods

### Requirements

In the process of designing the plugin, a number of requirements have been identified to assure the robustness of the plugin in a variety of usage scenarios.

The main scenario is the independent execution of a macro on each tile. This can be considered useful for quantifying tissue fractions, evaluating simple immunohistochemistry, etc. However, when looking for rare events like mitoses, objects falling on the boundary between adjacent tiles may be lost. This can be avoided by allowing some overlap between adjacent tiles. Another scenario is when the macro cannot be run in a totally independent way on each tile, for example when previously processed tiles are needed for some reason (backtracking, adaptive algorithms, etc). Finally, another scenario is when different magnifications should be processed.

The requirements that have been identified are the following:

- The plugin should be usable with different digital slide formats. Using Bio-formats as input library already allows for this, but robustness over internal parameters of different formats, such as series position or tile size, has also to be guaranteed. Thus, the plugin should contain also configurable parameters.
- The macro to be executed should be stored in a file, which position is not fixed but passed as a parameter.
- Even if the macro is independent from the image being processed, the developer should be able to recognize the coordinates of the sub-image itself.
- Overlap between adjacent tiles should be configurable through a parameter.
- Tiles extracted from the digital slide can be automatically deleted after macro execution or left in a temporary folder, according to user preferences. The latter possibility allows for their availability even after their processing.
- It should be possible to invoke the plugin with its parameters, from another macro too, to further automate execution.

### Experimentation

Three experiments have been made to demonstrate the effectiveness of SlideJ:

- To evaluate compatibility with common digital slide formats, we applied the plugin to all demo files hosted at the Openslide web site, which in turn are used to demonstrate Openslide features [18].
- Together with the plugin, we developed a number of example macros to demonstrate how SlideJ can be used for digital slide processing, starting from the typical problem of nuclear biomarkers quantification, with hematoxylin and diaminobenzidine (H/DAB) stained samples.
- The last experiment has been carried out to evaluate performance of the system according to tile size. For this, 10 digital slides from breast cancer core biopsies, immunohistochemically stained for progesterone, have been randomly selected from a previously available set. They were acquired with an Aperio CS scanner, at 20x magnification and thus with a spatial resolution of 0.5μm. At first, an attempt to open and process the slides as they are has been made, to evaluate how many could be directly processed with ImageJ, in spite of their size. Then, a macro has been repeatedly executed on each slide, by means of SlideJ changing tile size from 2048 to 16384 pixels per side to study the differences in performance, expressed as throughput (MPixels/minute). While we recognize that throughput heavily depends on the implemented algorithm, this experiment gives an idea to users on which tile sizes are better to address their implementations.

All experiments have been carried out on a personal computer configured in an almost typical way, though towards the high end: an Apple MacBook Pro with i7 processor, 4 cores at 2.2GHz, with 16GB of RAM and SSD disk. ImageJ v2.9.9-rc-43/1.51k, embedded in Fiji, has been set with 8192MB of maximum heap memory.

## Results

### Implementation

The SlideJ plugin has been implemented in Java, using Bio-formats as input library and the fiji-lib.jar for the interface. The latter is provided with the Fiji distribution of ImageJ, but it can also added to a standard ImageJ installation. It can be run on any operating system in which ImageJ can be run.

**Fig 1:**
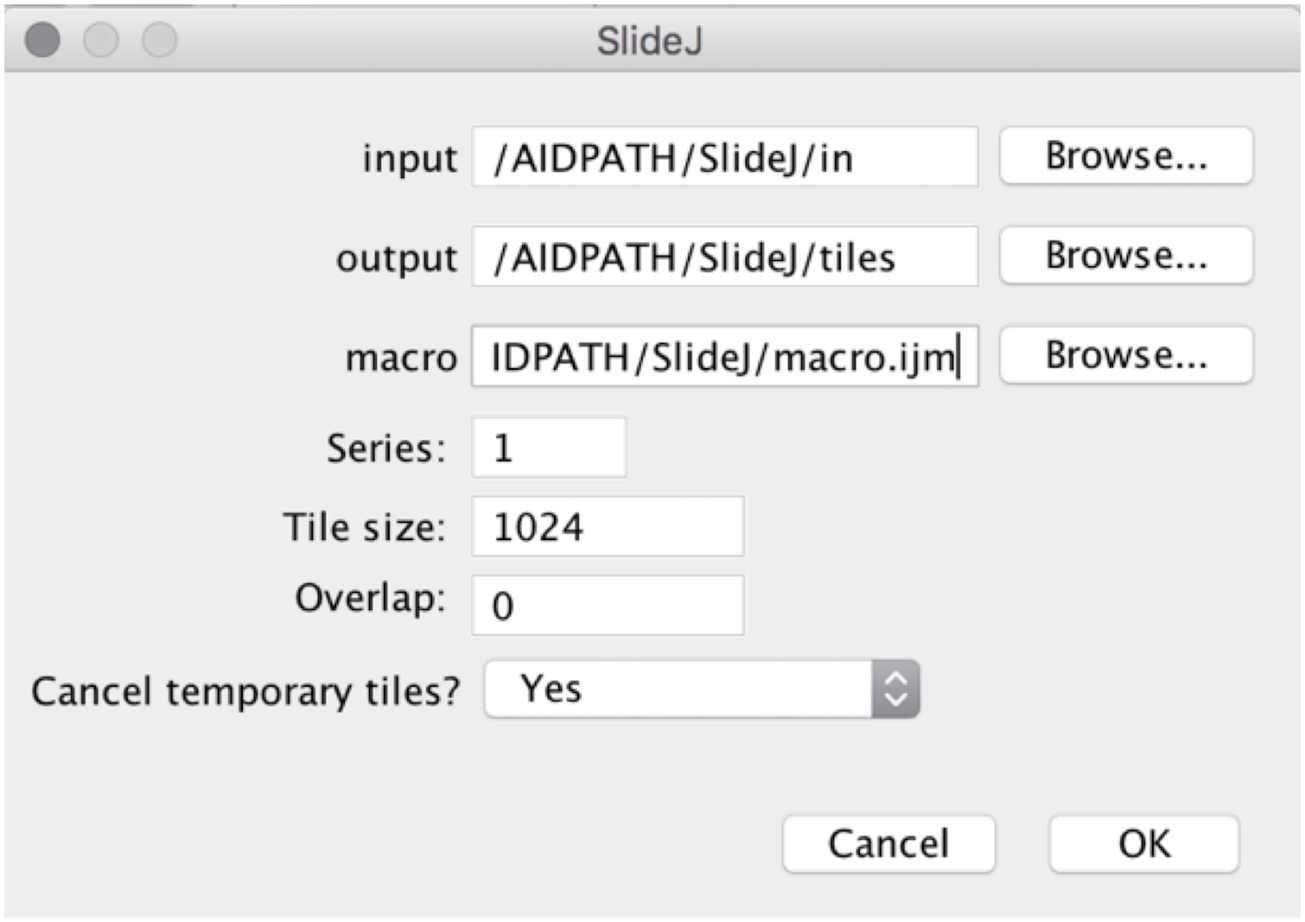
The SlideJ configuration dialog. Folder and file parameters can be selected by browsing the file system.

The interface of the plugin is a modal dialog (Fig 1) that resembles the one implemented in ImageJ for batch macro execution. The user is allowed to process multiple images present in a folder and store the eventual results in a different folder by setting up paths in the “Input…” and “Output…” fields, respectively. Since digital slide processing could be a time consuming task, the plugin allows the user to launch very long series of unsupervised tasks, which can be performed in free time (overnight or week end).

In the main dialog window, apart of input and output folders setting, a macro selection button allows the user to choose the file containing the macro to be executed on the tiles.

Most digital slide files are pyramidal, i.e., each resolution level is memorized as a tile series. However, the order in which series appear into the file is not the same for all formats. Thus, a “Series” parameter has been introduced to select the position in the file of the series that has to be processed (e.g., 1 for the highest resolution in SVS files).

Internal tile sizes are normally dependent on the system used for acquisition and are usually small (240x240, or similar sizes). The plugin instead provides a parameter to set the size according to user needs. Typical sizes will depend on the algorithm that has to be applied and on the memory available. In addition to that, another parameter defines the overlap between tiles, identical for X and Y directions.

The last parameter defines whether the tiles are deleted or not after each processing step. In fact, running the plugin, with this parameter set to “no”, and a void macro, produces a set of tiles that could be further used in some other processing environment, similarly to Deroulers method [14].

Tiles are stored in TIFF format with a file name that reflects their position on the overall digital slide according to the following template:

*<OriginalFileName*.*ext>*__*<series>*_*<X origin>*_*<Y origin>*.tif

Stored slides can then be reopened after their main processing step, since they are always identifiable (if not automatically deleted). This may enable multiresolution processing, backtracking algorithms, etc. The file name can be accessed also during processing, with techniques usual in ImageJ macro programming (i.e., reading and parsing the window title). This allows to know at any time which tile is being processed, in which series and of which slide. However, since those data are often useful, in provided macro examples we defined a function that returns that data.

The called macro should be designed to work on the opened image, and is responsible to close it and eventually any other window at the end of the execution.

One potential limitation is that each run of the macro on a tile is independent. Thus, in principle is impossible to pass variables from one step to the other. However, ImageJ allows to save data in its Results table, which provides persistence among tile processing steps, as demonstrated in the example macros.

### Compatibility

SlideJ has been tested on the files hosted on the OpenSlide demo site [18], and it successfully processed slides in the following formats: Aperio SVS, Leica SCN, Hamamatsu .NDPI, Mirax, Generic tiled TIFF and regular TIFF. Unfortunately Ventana/Roche .BIF format at present cannot be correctly opened with Bio-Formats, and thus neither with SlideJ.

### Example macros

Quantification of nuclear biomarkers in slides immunohistochemically stained with Hematoxylin and diaminobenzidine for peroxidase (H/DAB) has been taken as reference problem for the development of example macros. In fact, we at first developed a rough algorithm for that, based on colour deconvolution [19], then we also adapted ImmunoRatio, an ImageJ plugin that has been validated for that [20]. However, we did not aim at a robust implementation for clinical use, but just as demonstration of usage and functionality. Thus, the implemented macros do not discriminate normal and stromal tissue, and in-situ components.

In the macro examples we also implemented a set of functions to store variables or arrays. They can be used as they are or as the basis for more complex storage schemas. In Table 1 we list the macros developed for different H/DAB stained sample analysis.

**Table 1:** Example macros. File name and a short summary of macro content has been provided for each example macro.

File name	Description
*SlideJdemo1*.*ijm*	Simulates a basic evaluation of H/DAB positivity for each tile of each slide in the input folder. Its output is a Results table with a row for each processed tile, showing blue and brown area, and their positivity percentage.
*SlideJdemo2*.*ijm*	This macro is similar to SlideJdemo1.ijm, but it directly calculates the overall H/DAB positivity of each slide. Its output is a Results table with a row for each slide, showing blue and brown area, and their positivity percentage.
*SlideJdemo3*.*ijm*	This macro is used to demonstrate the use of more than one Results table to store data. It calculates H/DAB positivity using a modified version of the ImmunoRatio plugin, which outputs now not only an annotated image, but also a row in the Results table. This row is in turn read by the macro and stored in a differently named Results table.
*CallerDemo1*.*ijm*	It calls SlideJ with SlideJdemo1.ijm and then shows the tile with maximum H/DAB positivity. This example shows how to access previously stored tiles after a preliminary processing of the slide.
*CallerDemo2*.*ijm*	This is the macro used for performance tests. It calls SlideJ with SlideJdemo2.ijm macro, to enable batch mode before any other operation
*CallerDemo3*.*ijm*	It calls SlideJ with SlideJdemo3.ijm, to enable batch mode before any other operation for the sake of performance.
*SlideJfunctions*.*txt*	This file contains the same SlideJ specific functions used in the demo macros but put alone.

### Digital slide test set

**Fig 2:**
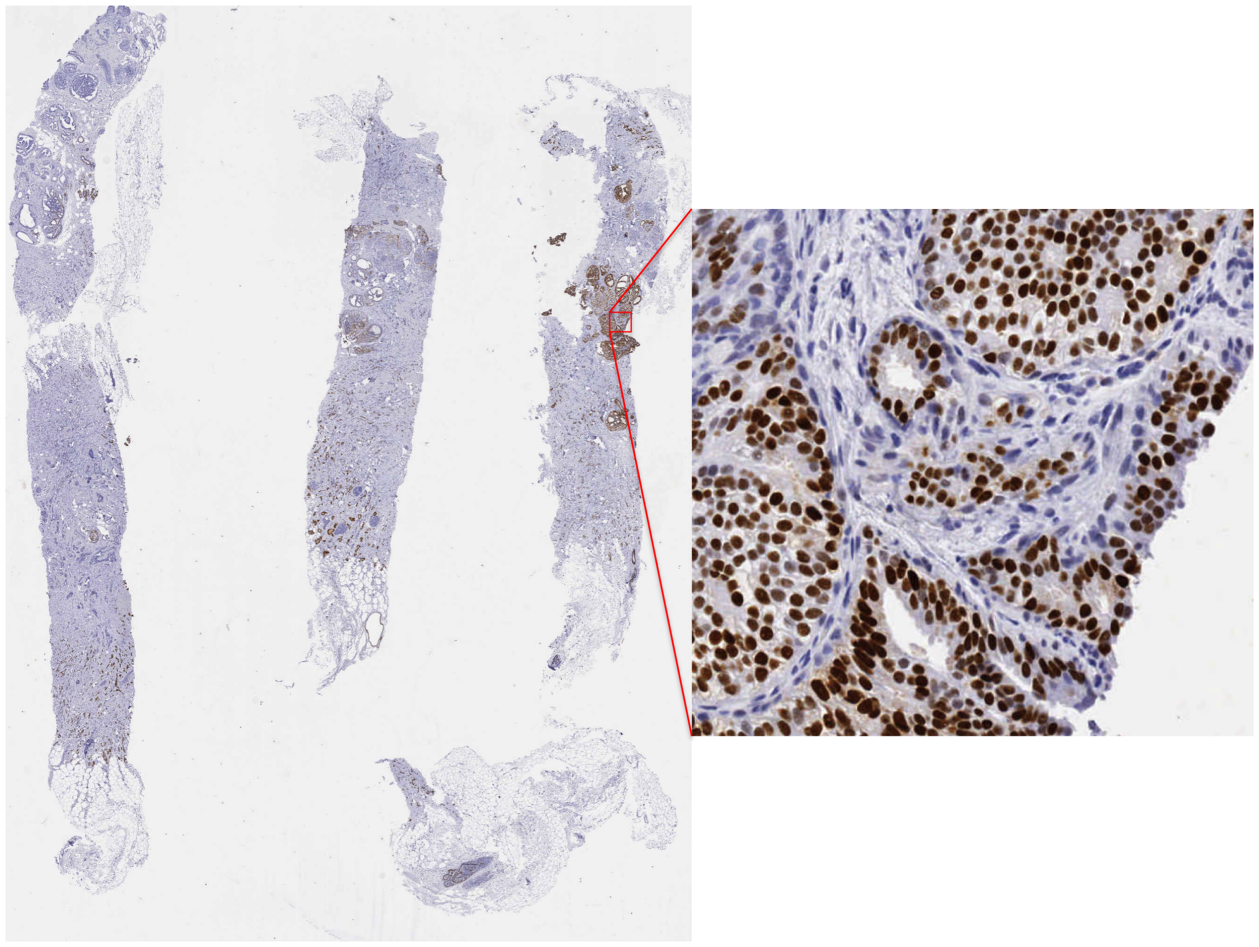
Sample slide. Overall view of a sample slide and one high magnification detail.

The ten digital slides for the test set ranged from 460Mpixel to 3.179 Gpixels. While not aimed at validation, the positivity calculated with the algorithm implemented in *SlideJDemo2* achieved a Pearson correlation of 0.93 with expert evaluated positivity. Fig 2 shows a sample slide.

A macro with the same algorithm of *SlideJDemo2*, but adapted to be applied to an entire slide, has been developed to check whether digital slides of the progesterone test set could be directly processed without SlideJ. In fact, it depends from the algorithm and from memory available to ImageJ, and thus we set our system for using a large amount of RAM for it (8GB). However, 6 out of 10 slides—the largest ones- could not be processed directly due to out of memory errors, as expected. This even if the implemented algorithm is very simple. With our memory setting, the threshold for exhausting memory was put around 700Mpixels, with degrading processing speed in slides slightly below threshold, likely due to swapping.

### Performance

**Fig 3:**
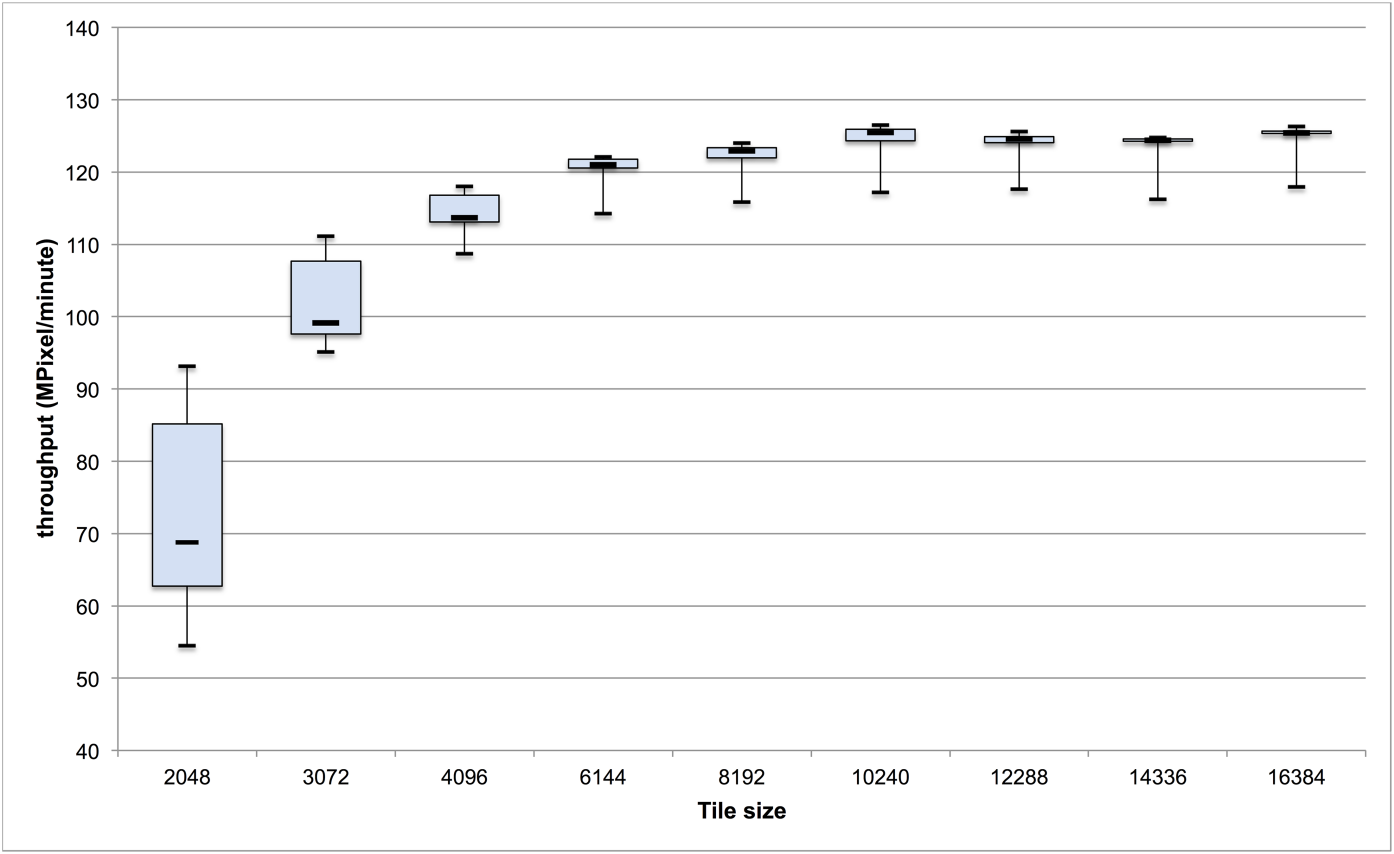
Performance of SlideJ execution. Throughput (MPixels/minute) by tile size is shown for the example macro SlideJdemo2, run in “batch mode” on series 1. Data is presented as box and whiskers plot (minimum, 1^st^ quartile, median, 3^rd^ quartile, maximum).

The *SlideJdemo2* macro, called from *CallerDemo2*, has been executed on the progesterone slide set, changing tile size in the following steps: 2048, 3072, 4096, 6144, 8192, 10240, 12388, 14336, 16384. Maximum throughput obtained was slightly above 125 Mpixel/minute. Fig 3 shows throughput for each tile size. No out of memory errors have been recorded during execution of SlideJ.

As it can be seen, smaller tile sizes result in slower processing, due to the overhead in accessing the original file and saving tiles. However, anything above 6144 pixels of tile size (that is, 36Mpixels tiles) is almost equivalent in terms of speed. In the experiment setting, at 6144 pixels of tile size, the average time needed for processing one slide is 9.5 minutes.

## Conclusion

Whole slide images cannot always be directly opened and processed with ImageJ and similar software, due to the need of being fully loaded in main memory, which is not often practically possible.

SlideJ provides a method to automatically process digital slides inside ImageJ. In principle, any digital slide format supported by Bio-formats now or in the future can be opened and processed using SlideJ. No specific workflow has been implemented, except the iterative loading of tiles from the digital slide. Processing and analysis are left to a macro written in the ImageJ scripting language, which is applied to each tile. No other plugins implement this function at present.

There are however some limitations. Persistence of partial results is implemented using Results Tables of ImageJ, which is a sort of workaround, although known to ImageJ users. Furthermore, at present we do not consider digital slides with more than one focal plane or time point. Finally, performance of macro-based processing is far from the needs of images as large as digital slides in production environments. However, the intended use of SlideJ is mainly rapid prototyping and testing of processing algorithms on digital slides aimed at research, that could then be translated in Java code or other implementations.

The plugin with Java source, examples macros and other programming examples can be found at the address https://github.com/MITEL-UNIUD/SlideJ as open source.
